# Hepatic angiomyolipoma with special attention to radiologic imaging

**DOI:** 10.1186/s40792-015-0038-0

**Published:** 2015-04-24

**Authors:** Kunitaka Kuramoto, Toru Beppu, Tomohiro Namimoto, Hiromitsu Hayashi, Katsunori Imai, Hidetoshi Nitta, Daisuke Hashimoto, Akira Chikamoto, Takatoshi Ishiko, Ken-Ichi Iyama, Osamu Ikeda, Yasuyuki Yamashita, Hideo Baba

**Affiliations:** Department of Gastroenterological Surgery, Graduate School of Life Sciences, Kumamoto University, 1-1-1 Honjo, Kumamoto City, Kumamoto 860-8556 Japan; Department of Multidisciplinary Treatment for Gastroenterological Cancer, Kumamoto University Hospital, 1-1-1 Honjo, Kumamoto City, Kumamoto 860-8556 Japan; Department of Diagnostic Radiology, Graduate School of Life Sciences, Kumamoto University, 1-1-1 Honjo, Kumamoto City, Kumamoto 860-8556 Japan; Department of Surgical Pathology, Kumamoto University Hospital, 1-1-1 Honjo, Kumamoto City, Kumamoto 860-8556 Japan

**Keywords:** Hepatic angiomyolipoma, Diffusion-weighted MRI, Apparent diffusion coefficient (ADC), Chemical shift imaging, Early venous return

## Abstract

**Background:**

Angiomyolipoma is a unique mesenchymal neoplasm composed of blood vessels as well as smooth muscle and adipose cells. The liver is a less common site of origin, and hepatic angiomyolipoma is often an incidental finding on diagnostic imaging or is identified on evaluation of nonspecific symptoms.

**Case presentation:**

We experienced four patients who were diagnosed histologically with hepatic angiomyolipoma. The preoperative diagnoses were angiomyolipoma in two patients, hepatocellular carcinoma in one, and cavernous hemangioma in one. Three patients were treated with hepatectomy (one laparoscopic and two open approaches), and the diagnosis was completed by histological investigation of the resected specimen. The remaining one was diagnosed from tumor needle biopsy. Diffusion-weighted magnetic resonance imaging (MRI) with respiratory triggering using *b* values of 0 and 800 s/mm^2^ was employed. An apparent diffusion coefficient map was generated from *b* values of 0 and 800 s/mm^2^ for calculation of the apparent diffusion coefficient. The apparent diffusion coefficient values were calculated as 3.66, 1.21, 1.80, and 0.91 in patients 1 to 4, respectively. In MRI imaging, fat component was clearly demonstrated with chemical shift imaging in three patients. Early venous return was detected in three patients with computed tomography angiography.

**Conclusion:**

Fat component and early venous return are important for a correct diagnosis of hepatic angiomyolipoma. Unfortunately, apparent diffusion coefficient values in hepatic angiomyolipoma were overlapping with those in other benign and malignant tumors.

## Background

Angiomyolipoma (AML) is a unique mesenchymal neoplasm consisting of blood vessels, smooth muscle, and adipose cells [1]. These neoplasms often arise in the kidneys [2], while the liver is a less common site of origin; hepatic AML is often an incidental finding on diagnostic imaging or identified on evaluation of nonspecific symptoms [3]. This tumor has been defined as a benign neoplasm, although it is suggested that some cases may have a malignant potential after hepatic resection [2,4,5].

Definitive diagnosis is based on pathologic findings supported by immunohistochemical staining. The smooth muscle cell component is the most specific to the diagnosis, and characteristically, these lesions stain positive for homatropine methylbromide-45 (HMB-45) and Melan-A [6,7]. Because hepatic AMLs are rare and have various imaging features that overlap with those of other tumors, definitive preoperative diagnosis is difficult to achieve, and more than half of the patients are misdiagnosed with hepatocellular carcinoma (HCC) or liver cell adenoma, which are much more common [8].

Magnetic resonance imaging (MRI) using conventional and diffusion-weighted sequences has been introduced as a valuable technique for the characterization of focal solid hepatic lesions [9-11]. Nowadays, the apparent diffusion coefficient (ADC) value has been introduced in quantitative measurements as an adjunct to diffusion-weighted MRI (DW-MRI) [10,11].

The relationship among clinical behavior and radiologic and pathologic features is not well characterized in this neoplasm [12,13]. There is a relatively small number of reports about resected hepatic AML from outside of China. The objective of this study was to characterize four Japanese patients with hepatic AML evaluated to determine radiologic characteristics by focusing on the DW-MRI findings.

## Case presentation

From 1997 to 2007, four patients were evaluated who were diagnosed histologically with hepatic AML in the Department of Gastroenterological Surgery, Kumamoto University Hospital. All clinicopathological data were collected in a prospective database. Radiologic data were independently assessed by two radiologists. All patients underwent ultrasonography (US), enhanced computed tomography (CT), CT angiography, and gadolinium-ethoxybenzyl-diethylenetriamine pentaacetic acid (Gd-EOB-DTPA)-enhanced or superparamagnetic iron oxide (SPIO)-enhanced MR imaging. To obtain a more correct preoperative diagnosis, we additionally performed DW-MRI with respiratory triggering using *b* values of 0 and 800 s/mm^2^. An ADC map was generated from *b* values of 0 and 800 s/mm^2^ for calculation of ADC.

The clinical characteristics of the patients are summarized in Table 1. Three patients treated with one laparoscopic [14] and two open hepatectomies were histologically diagnosed from the resected specimen and the remaining one from tumor needle biopsy. Only one patient had a symptom of abdominal discomfort. HBs-Ag was positive in one and HCV-Ab was negative in all patients. All tumors were solitary, and tumor size varied from 1.2 to 10 cm. Tumor markers were almost in a normal range, but one patient showed a high alpha-fetoprotein (AFP) level of 11.5 ng/ml (reference range <7 ng/ml) before hepatic resection. The preoperative diagnoses were AML in two patients, HCC in one, and cavernous hemangioma in one.

**Table 1:** **Clinical characteristics of the patients**

**Characteristics (** ***n*** **= 4)**	**Finding**
Gender (male/female)	3/1
Age (years)	72, 54, 51, 59
Symptom (positive/negative)	1/3
Diagnostic method (resection/biopsy)	3/1
HBs-Ag (positive/negative)	1/3
HCV-Ab (positive/negative)	0/4
Liver cirrhosis (positive/negative)	0/4
Child-Pugh (A/B)	4/0
Tumor diameter (mm)	12, 20, 22, 100
Number of tumor	All solitary
Tumor markers	
AFP (ng/ml)	2.7, 3.4, 3.9, 11.5
DCP (mAU/ml)	13, 24, 25, 28
CEA (ng/ml)	0.6, 1.0,1.3, 2.5
CA19-9 (U/ml)	1.0, 2.5, 3.3, 4.5

HBs-Ag, hepatitis B surface antigen; HCV, anti-hepatitis C virus antibody; AFP, alpha-fetoprotein; DCP, des-gamma-carboxyl prothrombin; CEA, carcinoembryonic antigen; CA19-9, carbohydrate antigen 19-9.

**Figure 1:**
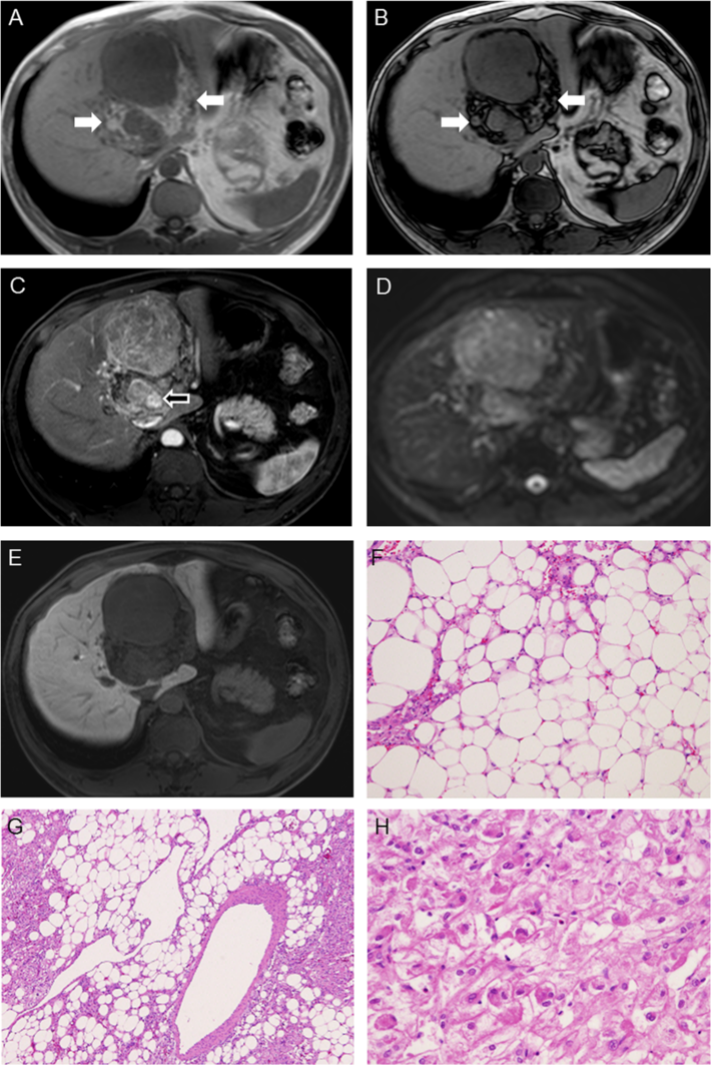
A case of Gd-EOB-DTPA-enhanced MRI findings of huge AML. In the T1W in-phase image **(A)**, the tumor itself is mixed intense in hypointense surroundings. In the in-phase hyperintense signal areas (white arrow), the loose signal in the T1W out-of-phase image **(B)** is consistent with the fat content **(F)**. In the arterial phase image **(C)**, a minor part of the tumor was remarkably enhanced (arrow) and this part is consistent with meandering blood vessels **(G)**. In DWI, **(D)** MRI displays an obvious hyperintense signal, whereas in the hepatobiliary phase **(E)**, it shows a homogenous hypointense signal. Major parts of the tumor are consistent with the smooth muscle cell component/components **(H)**, and these lesions were positive for HMB-45 and Melan-A.

**Figure 2:**
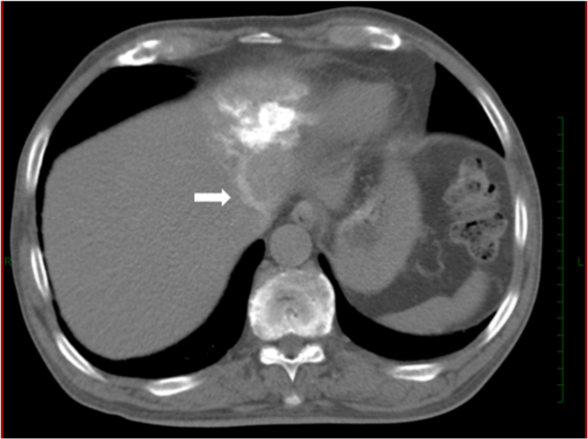
A case of AML with early venous return. CT angiography via the left hepatic artery showed a patchy enhanced tumor in S3 with early venous return to left hepatic vein (arrow).

Diagnostic features are summarized in Table 2. Early enhancement with delayed washout was clearly detected in three patients, but the tumor border was irregular without capsular formation (Figure 1). In MRI imaging, fat component was clearly demonstrated with chemical shift imaging in three patients. The ADC values were calculated as 3.66, 1.21, 1.80, and 0.91 in patients 1 to 4, respectively. Early venous return was detected in three patients with CT angiography (Figure 2).

**Table 2:** **Diagnostic imaging of the patients**

**Characteristics (** ***n*** **= 4)**	**Finding**
US - a heterogeneously hyperechoic mass (yes/no)	3/1
CT	
Plain CT - a heterogeneously low density mass with low attenuation value (< −20 HU) (yes/no)	2/2
Contrast-enhanced CT - early enhancement with delayed washout (yes/no)	3/1
Not round margin, the tumor border was irregular without capsular formation (yes/no)	1/3
Intratumoral vessels (yes/no)	2/2
Homogeneous enhancement (yes/no)	2/2
MRI	
T1-weighted imaging (hypo/hyper/mixed)	2/1/1
T2-weighted imaging (hypo/hyper/mixed)	0/1/3
Fat component in chemical shift imaging (yes/no)	3/1
ADC in diffusion-weighted imaging	0.91/1.21/1.80/3.66
Hypointensity in hepatobiliary phase on T1-weighted imaging (Gd-EOB-DTPA) (yes/no)	2/0
Intratumoral vessels (yes/no)	1/3
Homogeneous enhancement (yes/no)	2/2
Dynamic CT, CT angiography, or MRI early venous return (yes/no)	3/1

US, ultrasonography; CT, computed tomography; MRI, magnetic resonance imaging; ADC, apparent diffusion coefficient; Gd-EOB-DTPA, gadolinium-ethoxybenzyl-diethylenetriamine pentaacetic acid.

The diagnoses were confirmed histopathologically. As the components of the smooth muscle cells and adipose cell are specific to the diagnosis, HMB-45 was positive in all patients. Melan-A and αSMA, and Desmin are positive in four and three patients, respectively. There was no malignant AML. In three patients undergoing hepatectomy, the operation time was 2.8, 7.8, and 8.5 h and intraoperative blood loss was 50, 870, and 1,580 g, respectively. All four patients are alive without recurrence for 2.7 to 7.1 years.

## Discussion

Multiple modalities have been used to diagnose hepatic AML. Laboratory tests, viral markers for hepatitis, tumor markers, and liver function have not been proven to be specific or helpful in the diagnosis of hepatic AML. Based on the large studies of hepatic AML [1,13], only 23% to 50% of the patients could have been reliably diagnosed before the operation.

It is because variable imaging appearances are due to the varying proportion of three components: vessels, smooth muscle cells, and adipose tissue.

In this study, fat component was demonstrated by chemical shift MRI techniques in three patients. Identification of signal drop on fat-saturated T1-weighted sequences or opposed-phase chemical shift pulse sequences showed 100% specificity for the intratumoral fat [6]. It is well known that HCC sometimes showed a paradoxically high intensity in the hepatobiliary phase in Gd-EOB-DTPA-enhanced MRI [15]; conversely, hepatic AML never showed a high intensity. Early enhancement with delayed washout, mimicking HCC, was clearly detected in three patients, but the tumor border was irregular without capsular formation. Besides, early venous return in the arterial or portal phase was detected with various diagnostic imaging in three patients. Kassarjian et al. [16] reported a classification of hepatic hemangiomas with angiographic findings.

AML often contains a part of a hemangioma-like component inside the tumor. All three patients in this study demonstrated type 2 tumor, high-flow nodules, early filling of veins, and no visible direct shunts with no major vascular anomalies. We were able to identify early venous return in two patients only with CT angiography but in the remaining one with standard enhanced CT. The phenomena of early venous return might be useful in differentiation with the other hepatic tumors and caused early enhancement with delayed washout in the hemangioma-like component. Lately, an early draining vein has been reported to be seen in 73% of AML and was suggested to be useful for distinguishing AML from fat-containing HCC [12].

DW-MRI is sensitive to molecular diffusion and allows for tissue characterization by probing tissue microstructural changes [9,10]. We believe that examination of DW images in addition to routine abdominal MRI would enhance diagnostic performance of radiologists during evaluation of focal hepatic lesions.

Some studies [17,18] found that renal AML had significantly lower ADC than renal cell carcinoma, cysts, complicated cysts, and overall healthy parenchyma, which stated diffusion-restricting muscle and fat components as the causes for the decreased ADC of AML. Thus, we assessed ADC for liver AML. In this study, to obtain the true diagnosis of the tumor, we measured the ADC value as a quantitative measurement as an adjunct to DW-MRI [12]. We retrospectively examined 240 patients with 195 malignant (HCC, *n* = 137; liver metastases, *n* = 44; intrahepatic cholangiocarcinoma, *n* = 14) and 45 benign liver tumors (hemangioma, *n* = 37; focal nodular hyperplasia, *n* = 8). The mean ADC (×10^−3^ mm^2^/s) of malignant tumors was 1.19 ± 0.30; for benign tumors, this value was 1.98 ± 0.47. Unfortunately, the values of AML were overlapping with those of other benign and malignant tumors, and we could not find any differences between the Japanese patients and non-Japanese patients.

It has been reported that ADC measurements at three different diffusion gradients may be a complementary tool in the differential diagnosis of malignant and benign tumors [11]. More recently, it has been demonstrated that the ADC of the AML was significantly higher than that of fat-containing HCC (1.92 ± 0.29 × 10^−3^ mm^2^/s vs 1.33 ± 0.25 × 10^−3^ mm^2^/s, *p* < 0.001).

AFP is a well-known tumor marker of HCC. Preoperative AFP was positive in one patient who was diagnosed with HCC before hepatic resection. We have reported that preoperative AFP doubling time is a useful predictor of recurrence and prognosis after hepatic resection of HCC [19], but the AFP level in this patient did not increase before hepatic resection and continued to have an abnormal value postoperatively. Serial measurement is useful to distinguish nonspecific elevation of a tumor marker. In a patient without symptoms and risk factors for liver malignancy (such as chronic hepatitis B or C carrier, liver cirrhosis, alcohol abuse), with normal serum tumor markers and with imaging features suggestive of hepatic AML, conservative treatment with regular surveillance has been recommended [13]. Recently, a malignant AML in the liver was reported. To obtain a definitive diagnosis of a liver tumor mimicking AML, tumor needle biopsy is advocated. But if the tumor is located at the surface of the liver, laparoscopic exploration and biopsy is a preferred approach to avoid seeding of tumor cells.

## Conclusions

To obtain correct diagnosis of hepatic AML, evaluation of fat component with chemical shift imaging in MRI and early venous return in CT angiography is quite beneficial. Furthermore, in the era of laparoscopic surgery, laparoscopic liver resection is recommended for minimally invasive total tumor biopsy [20]. ADC has been recently reported as a useful marker to distinguish AML and other liver tumors [12]. Further prospective study comprising a large number of patients will be required.

## Consent

Written informed consent was obtained from the patients for the publication of this case report and any accompanying images.

## Abbreviations

ADC: apparent diffusion coefficient
AFP: alpha-fetoprotein
AML: angiomyolipoma
CT: computed tomography
DW-MRI: diffusion-weighted MRI
Gd-EOB-DTPA: gadolinium-ethoxybenzyl-diethylenetriamine pentaacetic acid
HBs-A: hepatitis B surface antigen
HCC: hepatocellular carcinoma
HCV: anti-hepatitis C virus antibody
HMB-45: homatropine methylbromide-45
MRI: magnetic resonance imaging
SPIO: superparamagnetic iron oxide
US: ultrasonography
